# Severe Bone Marrow Hypoplasia with Black Cumin *(Nigella sativa)* Ingestion in a Patient with T-ALL in First Complete Remission

**DOI:** 10.4274/tjh.galenos.2019.2019.0093

**Published:** 2019-08-02

**Authors:** Zehra Narlı Özdemir, Cemaleddin Öztürk, Işınsu Kuzu, Muhit Özcan

**Affiliations:** 1 Ankara University Faculty of Medicine, Department of Hematology, Ankara, Turkey; 2 Ankara University Faculty of Medicine, Department of Pathology, Ankara, Turkey

**Keywords:** Nigella sativa, T-cell acute lymphoblastic leukemia, Bone marrow hypoplasia

## To the Editor,

*Nigella sativa* L., commonly known as black cumin, black seed, or black caraway, contains the active component thymoquinone and has a historically extensive usage in traditional medicine. Most studies have focused on its beneficial effects and studies focusing on its possible toxicity are limited. To the best of our knowledge, this is the first report of an association between black cumin extract intake and myelosuppression.

**Figure 1:**
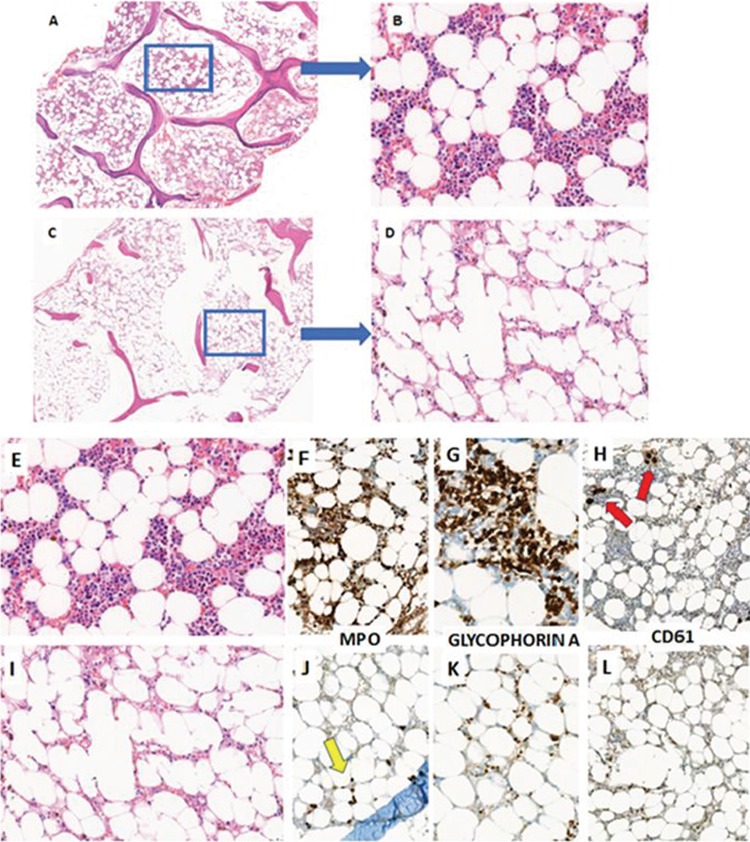
The bone marrow after treatment was normocellular in low (A) and high (B) power views. Following the *Nigella sativa* extract intake, a striking decrease in cellularity (C and D) was observed (H&E). The bone marrow biopsy following the treatment was normocellular (E) with dominant MPO-expressing myeloid lineage (F), increased nucleated glycophorin A-expressing erythroid precursors (G), and scattered megakaryocytes (red arrows) (H). Following *N. sativa* extract intake, within 2 months bone marrow biopsy showed severe decrease in cellularity (I). Seriously decreased number of MPO-expressing myeloid precursors (yellow arrow) (J), erythroid precursors (K), and vanished megakaryocytes (L) revealed that serious myeloid suppression was the cause of pancytopenia.

A 36-year-old man with T-cell acute lymphoblastic leukemia (T-ALL) in complete remission-1 (Figure 1) in a period with no maintenance treatment was admitted to our clinic with neutropenic fever and pancytopenia.

He had been diagnosed with T-ALL a year ago. A modified LINKER chemotherapy protocol was administered for T-ALL. Complete remission was achieved 3 weeks later and minimal residual disease (MRD) was negative by flow cytometric analysis. After 5 cycles of consolidation, 6-mercaptopurine (6-MP) and methotrexate (MTX) maintenance therapy was initiated orally. After 1 month the maintenance therapy was stopped because of toxic hepatitis. The clinical picture of toxic hepatitis was resolved 1 month after termination of the 6-MP/MTX treatment. Bone marrow aspiration and biopsy were performed to check the disease status and complete blood count results were normal at the same visit. The histopathological examination confirmed the presence of normocellular bone marrow with no MRD (Figure 1) and it was decided not to administer any medication for a while.

Two months later, the patient was admitted to our emergency department with neutropenic fever with severe pancytopenia. The clinical history of the last 2 months revealed intake of a milled powder of *N. sativa* seed in the amount of a teaspoon twice daily without any professional medical counseling. Laboratory tests at admission were as follows: hemoglobin, 8.1 g/L (reference range: 11.7-15.5 g/dL); leukocytes, 0.6x10^9^/L (4.5-11x10^9^/L); neutrophils, 0.08x10^9^/L (1.8-7.7x10^9^/L); monocytes, 0.02x10^9^/L (0.2-0.95x10^9^/L); lymphocytes, 0.48x10^9^/L (1.5-4x10^9^/L); reticulocytes, 0.0024x1012/L (0.2-0.16x1012/L); platelets, 4x10^9^/L (150-400x10^9^/L); serum vitamin B12, 1211 pg/mL (126-505 pg/mL); and serum folic acid, 19.75 ng/mL (5.9-24.8 ng/mL). The blood film was compatible with aplasia without any signs of blastic infiltration. Quantitative PCR for serum parvovirus B19, CMV PCR, and HIV serology were negative and serum EBV VCA IGG/IGM and haptoglobin levels were in the normal ranges. There was no infectious explanation for pancytopenia. No paroxysmal nocturnal hemoglobinuria clones could be detected. The patient used *N. sativa* L. called “çameli”, the only black cumin seed cultivated in Turkey, which is smaller than seeds planted in other Mediterranean countries, the Middle East, and North Africa. The active component of *N. sativa* and the blood level of the molecule were not analyzed since the patient had not taken black cumin for a week.

Bone marrow aspiration and biopsy were performed. On microscopic examination the bone marrow was hypocellular with suppression of all cell lineages, without any obvious infiltrative pathology (Figure 1). For supportive therapy, granulocyte colony-stimulating factor together with transfusion with packed red blood cells and platelets were administered. The patient’s neutrophil and platelet counts returned to normal on days 15 and 17, respectively, and after stopping *N. sativa* ingestion values were as follows: hemoglobin, 13.2 g/L; leukocytes,: 5.6x10^9^/L; neutrophils, 3.0x10^9^/L; monocytes, 0.5x10^9^/L; lymphocytes, 2.0x10^9^/L; platelets, 162x10^9^/L. The 6-MP and MTX maintenance therapy was completed with a dose reduction. He was in complete remission with no MRD at the last follow-up visit.

Thymoquinone is the best known component of *N. sativa* and has cytotoxic and immunosuppressive effects on cancer cell lines [1,2]. In rats, *N. sativa* oil induced about a 2-fold decrease in antibody production in response to typhoid vaccination as compared to the control group and a particularly significant decrease in neutrophil counts was demonstrated [1]. A study performed by Swamy and Tan [3] showed cytotoxic and immunopotentiating effects of an ethanolic extract of *N. sativa* seeds on different types of cancer cell lines. Zaoui et al. [4] reported alterations in hemoglobin metabolism and decreased leukocyte and platelet counts due to chronic exposure to the fixed oil of *N. sativa* in rats. The results of these studies support the suppressing effect of *N. sativa* seeds on bone marrow cells due to the various effective chemicals in its content [5].

Although there is lack of evidence about their advantages and disadvantages, complementary and alternative medicines are becoming popular. Ingesting herbal medicines or extracts can cause drug/drug and drug/herb interactions. Although the US Food and Drug Administration classified *N. sativa* seeds as “generally recognized as safe”, the sphere of influence of *N. sativa* seed extracts on bone marrow has not been elaborated and still remains unknown. This is the first presented case of *N. sativa*-induced pancytopenia and the myelosuppressive effect of black cumin is worthy of further investigation.
